# Understanding Implementation of the Family and Community Nurse Role Using Consolidated Framework for Implementation Research: A Case Study

**DOI:** 10.1155/nrp/8734311

**Published:** 2026-02-19

**Authors:** Erica Busca, Erika Bassi, Simona Milani, Dino Stefano Di Massimo, Miriana Miotto, Stefano Tosatti, Luana Zampelli, Susan M. Jack, Alberto Dal Molin

**Affiliations:** ^1^ Department of Translational Medicine, University of Piemonte Orientale, Novara, Italy, unipmn.it; ^2^ Health Professions Directorate, Maggiore Della Carità Hospital, Novara, Italy; ^3^ Health Professions Directorate, Local Health Authority of Biella, Ponderano, Italy; ^4^ School of Nursing, McMaster University, Hamilton, Canada, mcmaster.ca

**Keywords:** Consolidated Framework for Implementation Research, family and community nurse, implementation process, organizational factors, primary health care, qualitative research

## Abstract

**Aim:**

To explore the factors influencing the Family and Community Nurse (FCN) role implementation within the local health authority (LHA) of Biella, Italy.

**Design:**

Qualitative single embedded case study.

**Methods:**

Twenty‐two participants (18 FCNs and 4 managers) were interviewed using in‐depth semistructured interviews and timeline elicitation. Observational data were recorded in field notes. Data were analyzed using the Framework Analysis approach and organized through the Consolidated Framework for Implementation Research.

**Results:**

At the outer setting level, alignment with national policy and population needs supported adoption, although public awareness of the role was initially low. Within the inner setting, organizational flexibility enabled role adaptation, especially regarding project participation and time allocation. However, fragmented coordination with existing services and the persistence of task‐based workflows limited integration. At the individual level, FCNs expressed strong motivation and professional identification, but also reduced confidence linked to limited preparation for community‐based and educational activities. The process was supported by reflective leadership and the progressive tailoring of strategies to local needs and staff competencies.

**Conclusion:**

Implementing the FCN role is a dynamic process shaped by structural conditions, professional boundaries, and stakeholder engagement. Sustainability requires system‐level support, cross‐sector collaboration, and a clearly defined scope of practice.

**Impact:**

This study highlights key organizational and contextual conditions required to support the sustainable integration of FCNs. These insights may guide health service managers and policymakers in strengthening community‐based care models, improving interprofessional coordination, and investing in role‐specific education to meet the needs of aging populations.

**Reporting Method:**

The study adhered to the Standards for Reporting Qualitative Research.

**Patient or Public Contribution:**

None.

**What Does This Paper Contribute to the Wider Global Clinical Community?**
• Offers one of the empirical applications of the Consolidated Framework for Implementation Research (CFIR) to explore the introduction of the FCN role in a European context, demonstrating how multilevel determinants interact dynamically during real‐world implementation.
• Identifies the enabling role of organizational flexibility and leadership in tailoring implementation strategies to local workforce capacities and community needs, thereby supporting the transition from task‐oriented practice to proactive, community‐centered care.
• Highlights how the development of FCNs’ clinical leadership capabilities is essential for consolidating their role in community‐centered care, underscoring the need to strengthen these competencies alongside technical preparation.

## 1. Introduction

An aging population and the increasing rates of chronic degenerative diseases and disabilities result in substantially more demands on the healthcare system. In Italy, data from the Italian National Institute of Statistics (ISTAT) indicate that 32.3% of citizens (> 65 years) have chronic conditions and comorbidities, with the proportion of people over 80 with chronic conditions doubling between 2001 and 2020 [1]. These trends extend beyond Italy to the Organization for Economic Co‐operation and Development (OECD) countries, where, by 2050, the proportion of individuals over 65 is projected to reach 29%, with more than 60% expected to have one or more chronic conditions [2]. In addition to chronic conditions, frailty is also increasing. A recent meta‐analysis of more than 1.7 million adults across 62 countries estimates a frailty prevalence of 16% among individuals aged 60–69 years, rising to 51% in those over 90, with variations depending on the measurement scale used [3]. Recent Italian evidence, adopting a bio‐psycho‐social approach to frailty [4], reports a prevalence of 39.8% among community‐dwelling older adults, with higher proportions observed among women and those in the oldest age groups [5]. Taken together, the combined trends of population aging, multimorbidity, and frailty highlight increasing demand for health and social care services. The COVID‐19 pandemic further exposed the inadequacies of healthcare systems, particularly in responding to the needs of vulnerable populations—such as older adults with chronic conditions [6]. Therefore, rethinking how health systems are designed and delivered has become a strategic priority. Increasing attention is being paid to prevention, continuity of care, and improved access to services in community and home‐based settings. Policymakers and healthcare professionals are advocating for integrated, person‐centered, community‐based models tailored to the needs of individuals with chronic conditions and their families [7]. In this evolving context, primary care nursing roles—such as the Family and Community Nurse (FCN)—are emerging as key contributors to the shift toward proactive, community‐oriented care.

## 2. Background

The World Health Organization (WHO) advocates for the FCN role to strengthen health systems and meet the evolving care demands of aging populations facing an increased burden of chronic conditions. FCNs are registered nurses with specialized training who operate at the intersection of individual, family, and community care. Their responsibilities typically include delivering primary healthcare, promoting health, preventing illness, managing chronic conditions, and coordinating care across health and social services [8]. In Italy, the FCN role had already been piloted in several regions prior to its formal recognition [9]. Ministerial Decree No. 77/2022 [10] subsequently institutionalized the role and called for the functional activation of new structures, such as Community Health Centers and Territorial Operational Centers. This Ministerial Decree positioned the FCN as a central figure in the reorganization of primary care. However, due to the decentralized nature of the Italian health system—comprising 128 local health authorities (LHAs) [11]—the responsibility for adopting, hiring, and utilizing FCNs was delegated to each LHA at the regional level, resulting in considerable variation in how the FCN role was implemented. In some LHAs, existing district nurses were designated as FCN role without major changes to their scope of practice. In other LHAs, FCNs were introduced as a distinct role with a focus on prevention and health education at the individual, family, and community levels, while district nurses or domiciliary nurses continued to provide direct care (e.g., wound management) [12]. Although flexibility in adapting the FCN role to local community needs is essential, this has led to inconsistent implementation nationwide and a lack of clarity at organizational and community levels regarding the distinct functions of FCNs compared to other community‐based nursing roles [13]. These inconsistencies are further reflected in disparities in care delivery standards, with a deviation from the recommended FCN‐to‐inhabitant ratio (1:3000) [10] and actual ratios averaging around 1:16,000 [14]. This variability is not unique to Italy; the introduction of the FCN role within multidisciplinary community and primary healthcare models has also been uneven in other European contexts [12], reflecting broader challenges in standardizing advanced nursing roles within diverse healthcare systems.

The transition to and implementation of a new model of community‐care nursing is complex and involves interactions between multiple interconnected system components, including organizational structures, healthcare providers, and community needs [15]. Evidence suggests that organizational efforts to implement such changes often fall short of their intended outcomes [16]. Barriers to successful implementation can arise at various levels, including individual practitioner behaviors, organizational processes, and broader policy frameworks [17]. At the individual level, limited educational opportunities and misalignment between training programs and the competencies required for advanced roles can create significant challenges [18]. At the team level, general practitioners often lack a clear understanding of the distinct functions and responsibilities associated with advanced practice nursing roles, leading to role ambiguity and resistance to integration within care teams [19]. Organizational barriers, such as role and responsibilities redefinition further hinder integration. Moreover, shortages of staff, administrative support, and essential equipment exacerbate these difficulties [20]. In contexts where FCNs have been implemented, supportive leadership and clear policies have proven critical to overcoming these barriers [13].

Given that the success of advanced practice nurse role implementation is highly influenced by contextual factors, it is essential to understand the local barriers and facilitators that have served to influence the integration of FCN in primary care. Nilsen and Bernhardsson argue that regional variations within healthcare delivery models, organizational structures, and policy frameworks significantly affect how APN roles are integrated and sustained [21]. Therefore, a nuanced understanding of local factors is critical to tailoring strategies that promote the effective adoption and utilization of FCNs across diverse settings in Italy. Despite the growing interest in FCNs as a solution to workforce and care delivery challenges in Italy, empirical research on their implementation remains limited. Given the unique geography, political contexts, and healthcare systems across Italy’s LHAs, the purpose of this study was to explore and describe the factors influencing how the FCN role was implemented within one LHA located in Northern Italy—the Azienda Sanitaria Locale of Biella. The overarching research question was as follows: How was the FCN role implemented within the Biella LHA, and what contextual, organizational, and professional factors shaped this process over time?

## 3. Methods

### 3.1. Study Design

A qualitative single embedded case study design was conducted to describe the process of implementing the FCN role into the real‐world context in which it occurred, a single LHA in Northern Italy. This design was chosen due to its suitability for answering “how” and “why” questions related to phenomena within its real‐world context, especially when the boundaries between phenomenon and context may not be clear [22].

#### 3.1.1. The Case

In a qualitative case study, it is important to define and bind the case. In this study, the “case” or social process being studied was the implementation of the FCN role, where implementation is defined as the critical transition between an organization’s decision to adopt an intervention and the consolidated use of that intervention [23]. The case was then bound by the study setting and time frame corresponding to the ongoing implementation of the role. The study setting was a single LHA in Northern Italy. The LHA of Biella consists of a single district where nine nursing teams, including FCNs and district nurses, operate. Each team is responsible for a specific geographical area, with their operational headquarters located in the Community Health Centers.

### 3.2. Theoretical Framework

The Consolidated Framework for Implementation Research (CFIR) [24] guided data collection and data analysis. The CFIR provided a comprehensive structure to examine multilevel determinants of implementation across five domains: outer setting, inner setting, intervention characteristics, characteristics of individuals, and implementation process.

Three propositions were selected a priori to guide the study’s focus [22], informed by the CFIR assumptions [24]: (1) changes in the external context (outer setting) influence implementation of the FCN role, often acting through the organizational environment (inner setting); (2) individuals actively shape the implementation process through their roles and decisions (individuals); and (3) successful implementation typically requires a multilevel active change process, encompassing individual to organizational transformations (implementation process).

### 3.3. Participants

The triangulation of data types and sources is a hallmark characteristic of case study research [22]. Two distinct data sources, which could provide rich descriptions of the implementation process and context, were purposefully sampled and invited to participate in this study: (1) FCNs and (2) program managers. The inclusion criteria for the invited nurses were as follows: (1) completion of a postgraduate degree, a Master in FCN; (2) employment as an FCN within the LHA; (3) direct involvement in delivering care within this role at some point between 2017 and 2024; and (4) a willingness and ability to share detailed descriptions of their experiences, in Italian. Within this organization, the full population of middle and senior managers with any role or responsibility related to the supervision of FCNs or implementation and integration of this model of nursing care within the LHA was also invited to participate. A purposive criterion sampling strategy was therefore adopted: Only individuals meeting these criteria were invited (*n* = 22), and all agreed to participate. Participants were asked to retrospectively reflect on their experiences implementing this role from the time of their training in FCN to the time of the interviews in 2024. Because some FCNs had already begun performing tasks aligned with the FCN role before its formal organizational adoption, participants were asked to reflect on both their own professional transition and the progressive implementation of the role within the Biella LHA. This approach allowed for the identification of how professional transitions at the individual level and organizational changes within the LHA unfolded concurrently and influenced one another over time.

### 3.4. Data Collection

To ensure a rich and comprehensive understanding of the case, data were collected using multiple data types which were used to corroborate and contextualize findings [22]. Data collection techniques included observation and face‐to‐face semistructured one‐to‐one interviews augmented with the completion of timelines as a data elicitation strategy.

Observational data were collected over a 30‐day period. In the observer‐as‐participant role [25], the primary qualitative researcher (EBu) immersed herself in the daily activities of the FCNs. This immersive approach facilitated a comprehensive understanding of how FCNs organize their work, prioritize activities, and navigate their professional responsibilities. In addition to gathering insights about the context in which FCNs work and how work is organized, this observational period also served to build trust and rapport with the FCNs prior to conducting interviews. This approach proved beneficial in several ways: It fostered a comfortable atmosphere during interviews, encouraged open and candid discussions, and provided a shared context that could be referenced to enrich the interview data.

All participants were invited to complete a single semistructured interview. The focus of the interview was to collect information on participant experiences of either implementing the FCN role within the organization or delivering care as an FCN, perceptions of the factors influencing the adaptation and implementation processes (and when they served as either barriers or facilitators), challenges encountered, and the strategies used to address these challenges, in the implementation process, and methods of evaluation (Table 1). Concepts explored in the interview, and the questions posed, reflected key constructs from the CFIR. The interviews were conducted in Italian by a nurse researcher (EBu), with extensive content expertise in nursing care delivery models and family and community nursing and substantive experience in conducting qualitative interviews. All interviews were conducted face‐to‐face; however, participants were given the option to identify the location for the interview. Participants were informed that the interviews would last up to 1 hour in length. At the start of each interview, participants were also guided to construct a timeline documenting significant milestones and challenges, mapped along with their recollection of the implementation process. This form of graphical elicitation enhanced participant reflection and provided a clearer understanding of key implementation events from their perspectives [26]. All interviews were audio‐recorded. Copies of each timeline were made and kept by the researcher. Demographic data were also collected. Following each interview, field notes were maintained to document information about the physical location of the interview, key concepts discussed and expanded upon, and impressions of the participant’s engagement and emotional reactions during the interview.

**TABLE 1:** Sample questions used in the semistructured interviews.

Questions asked to managers
What were the key milestones that marked the local adaptation of the FCN model in your context?
* How were decisions made and by whom?* (Process)
How did contextual factors (outer setting) influence the implementation process?
* What external pressures were present?* (outer setting)
How was the integration of the FCN role envisioned alongside existing services and professionals?
* How did professionals respond to this new role?* (inner setting)
What strategies were adopted to overcome implementation challenges?
* Can you describe whether they emerged from formal planning processes or evolved more informally in practice?* (Process)

**Questions asked to FCNs**

What were the key steps you experienced in becoming a FCN?
* How did your tasks or responsibilities change?* (process)
How did contextual factors influence the way the FCN role was implemented and adapted?
* How did local needs or population characteristics impact your work?* (outer setting)
How did your role relate to existing services (e.g., home care)?
* How did the organization influence the coordination of your role with existing services?* (inner setting)
What kind of support did you receive in your role?
* What aspects of your background or training supported you, and in what areas did you feel less equipped?* (characteristics of individuals)

### 3.5. Data Analysis

The data were systematically analyzed using the framework analysis approach [27] explicitly guided by the CFIR. Interviews were transcribed verbatim in Italian with identifying information removed. A team of two researchers then familiarized themselves with the content through repeated reading and conducted the coding process by identifying key segments of text. Drawing on CFIR as an a priori analytical structure, an initial coding framework was developed in which each code was mapped onto one of the CFIR domains and, when applicable, to specific constructs within these domains. No data were found that fell outside the scope of the CFIR domains or constructs, and therefore, no inductive refinement was required. A spreadsheet was used to generate a matrix, where the data were charted by summarizing each transcript’s content according to the CFIR domain and construct. This matrix facilitated systematic comparison across participants and across domains of the framework, supporting an integrated interpretation of how multilevel factors influenced implementation. While immersed in the data, ideas and concepts were discussed within the wider research team to support reflexive interpretation and theme development. The timelines were analyzed in terms of content to enrich or confirm the categories that emerged from the interview analysis and to understand the temporal sequence of events. For reporting purposes, selected quotations were translated into English and discussed within the research team to ensure conceptual equivalence and interpretive coherence.

### 3.6. Rigor and Reflexivity

Multiple strategies were employed to promote the overall rigor of the study findings. Data source, type, and researcher triangulation were used to support the credibility of the findings. The involvement of multiple researchers with methodological expertise in applied qualitative research (EBa, EBu, and SMJ) and content expertise with respect to community nursing (EBu, SMJ) in the data analytic process contributed to a consistent and transparent approach to coding and theme development. The researcher’s positionality in the field, in an observer‐as‐participant role, was addressed through reflexive note‐taking and regular team discussions. An audit trail of research decisions was maintained to support transparency regarding how field immersion could have shaped analytic interpretations.

### 3.7. Ethical Considerations

The study was approved by the Local Ethics Committee (Approval No. CE 104/2024). Informed consent was obtained from all participants prior to their involvement in the study. Confidentiality and anonymity were ensured by assigning each participant a unique alphanumeric identification code and removing any identifiable information from the transcripts.

## 4. Results

A purposeful sample of 22 individuals, 18 FCNs and 4 managers, who were strongly positioned to describe the process, successes, and challenges of implementing the FCN role within a single LHA in Northern Italy, were interviewed. The FCNs worked in seven of the nine nursing teams within the district of the LHA. Most participants identified as female (*N* = 18), with a mean age of 49 years (SD ± 7.5). Participants reported an average of 24.7 years of overall work experience (SD ± 8.6) and relatively recent experience working as FCNs, averaging 4 years in the role (SD ± 3.8, median 1.8, range [0.7–12]).

The multilevel factors perceived by participants that influenced the implementation of the FCN role within this LHA were systematically organized using CFIR constructs [24]. The analysis yielded 45 distinct factors distributed across four of the CFIR domains. Information on directionality, or whether a factor positively facilitated (↑) or negatively impeded (↓) the implementation process, was also elicited. The implementation of the FCN was facilitated by work structures (e.g., hierarchical organization), individual competencies aligned with role responsibilities, and dedicated time for reflection, debriefing, and information sharing within the team. Conversely, barriers stemmed from external contextual factors (e.g., local conditions) and challenges in integrating the role within district nursing care. The persistence of preexisting workflows designed for individual‐level care rather than community‐oriented care further hindered the transition. Additionally, the introduction of proactive healthcare models highlighted fragmented communication between organizational structures, limiting relational connections between hospital departments and the district. In Table 2, a detailed synthesis of these findings is summarized, demonstrating the interplay between various contextual, organizational, and individual‐level influences, providing insights into the mechanisms that influenced FCN implementation.

**TABLE 2:** Barriers and facilitators identified for each domain and construct.

Construct	Factor	Directionality
Domain: Outer setting		
Local attitudes	Lack of FCN role understanding by citizens Culture of immediacy prioritizes short‐term needs Overcoming stereotypes of district nurses	↓ ↓ ↑
Partnerships and connections	Connections with community‐based organizations	↑
Policies and law	Legislation (Ministerial Decree 77/2022)	↑
Local conditions	Aging population Territorial morphology	↓ ↓

Domain: Inner setting		
Structural characteristics: physical infrastructure	Colocation of health and social services in CHC Lack of adequate infrastructure in the CHC Lack of signage in TOCs	↑ ↓ ↓
Structural characteristics: work infrastructure	Adoption of the primary nursing delivery of care model Workplace mentoring Generational renewal of GPs Hierarchical FCN dependence on the Healthcare Professions Directorate	↑ ↑ ↑ ↑
Access to knowledge and information	Access to continuous education programs	↑
Relational connections and communication	Structured information sharing within nursing teams Interprofessional collaboration Organizational silos	↑ ↑ ↓
Relative priority	Establishment of TOCs Task‐oriented care	↑ ↓
Incentive systems	Lack of financial incentives	↓
Available resources	Staff allocation	↑

Domain: Individuals		
Capability	Work experience Assessment skills Leadership style	↑ ↑ ↑
Opportunity	Organizational autonomy Proximity to workplace	↑ ↑
Motivation	Motivation for professional development Motivation for role transition	↑ ↑

Domain: Implementation process		
Teaming	Lack of recognition for FCN community‐based initiatives within the team Creating a cohesive group	↓ ↑
Reflecting and evaluating	Structured reflection spaces for improvement Monitoring system for community projects Benchmarking across neighboring organizations	↑ ↑↓ ↑
Adapting (integrating community interventions into the nursing workflow)	Workload complexity Day‐to‐day scheduling Extratime commitments	↓ ↓ ↓
Engaging	Staff engagement (FCN and health professionals) Patient‐community engagement	↑↓ ↓
Tailoring strategies	Advanced scheduling of community events Flexible work shifts Mandatory participation in community events Selection of FCNs assigned to the TOCs Formal and informal meetings	↑ ↑ ↓ ↑ ↑

*Note:* Directional arrows indicate whether each factor was perceived by participants as facilitating (↑) or hindering (↓) the implementation of the FCN role.

Abbreviations: CHCs, Community Health Centers; FCNs, Family and Community Nurses; GPs, General Practitioners; TOCs, Territorial Operational Centers.

The findings section is organized into four parts: (1) a description of the implementation process over time and the strategies adopted to address emerging challenges; (2) the main outer setting factors; (3) key inner setting factors; and (4) the individual‐level factors influencing the adoption of the FCN role.

### 4.1. Implementation Process

**FIGURE 1:**
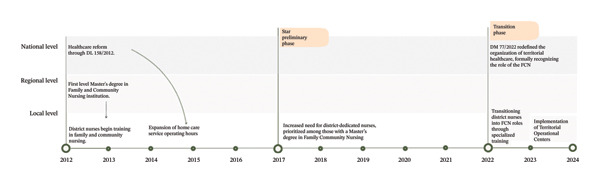
Implementation process timeline.

There was consensus across participants that two critical contextual events, both occurring in 2012, were the primary driving factors prompting LHAs to transition to a Family and Community Nursing model at local levels. These were as follows: (1) the introduction of Master’s degree programs in Family and Community Nursing and (2) the enactment of the Decree‐Law (DL) 158/2012. This legislation restructured community healthcare by expanding home care service hours, fostering the development of multidisciplinary teams, and strengthening integrated primary care to enhance accessibility and continuity of care (Figure 1).

In Biella LHA, discussions regarding the formal adoption of the FCN role began in 2017. The period from 2017 to 2022 represents a preliminary phase of FCN implementation. During this time, the expansion of home care service operating hours necessitated an increase in nursing personnel. In response, managers reassigned nurses to district services, prioritizing those who had completed the FCN Master’s degree (Table 2_Available resources). Although these nurses were not initially designated as FCNs in this new role, their specialized education introduced a paradigm shift in care models. In this novel role, they moved beyond the historically task‐oriented model to a model that prioritized patient and family‐centered approaches, community‐based interventions, and interdisciplinary collaboration. Despite the recognition of their advanced nursing training and its use as a criterion for workforce allocation, their role remained poorly understood by managers, limiting their full integration into the system in these early phases. District nursing managers struggled to communicate the significance of this transition, and as one manager explained: “*At those times, it was not easy. It was difficult to explain, even to the other nurse managers, the importance of what was happening*” (M_4).

In response to these challenges, and as an innovative initiative to increase the visibility of their role and promote understanding of the novel health promotion components of FCN practice, some FCNs proactively implemented community‐based interventions. On a voluntary basis, they organized health education sessions in schools, facilitated citizen meetings on fall prevention, and raised awareness about heatstroke risks. These initiatives not only strengthened their connection with local communities but also revealed the need for a more structured integration of community‐based activities into routine FCN practice. While these voluntary efforts demonstrated the potential impact of FCNs in public health promotion, they also highlighted the challenge of sustaining and formally integrating such initiatives into the broader healthcare framework.

The Year 2022 marked a pivotal turning point in the full adoption and integration of FCNs within this district’s healthcare services. This transformation, catalyzed by the Ministerial Decree 77/2022, formally and legally redefined the organization of community healthcare across Italy, by effectively mandating that LHAs adopt this model for care delivery. Crucially, the decree formally recognized the FCN as a central professional role within the healthcare system, underscoring its widespread presence and essential role in delivering integrated, community‐based services that advanced health promotion and disease prevention.

To ensure the effective implementation of the Ministerial Decree 77/2022 and facilitate the systemic expansion of FCNs, nurse managers adopted a strategic approach. This involved transitioning existing district nurses to FCN roles following specialized training. Additionally, hospital‐based nurses seeking to transfer to work in one of the new community‐based FCN roles were subsequently mandated to hold the specialized Master’s degree in Family and Community Nursing. This policy not only reinforced the professionalization of FCNs but also aligned workforce development with the evolving priorities of the LHA. As one manager emphasized, there was a clear organizational vision: “*With our top leader, we always pursued this goal: all district nurses should be Family and Community Nurses*” (M_1).

The transition phase was characterized by a collaborative approach, with managers actively involving FCNs from the early design stages to ensure the new role aligned with community needs and addressed regulatory uncertainties (Table 2_Engaging). This inclusive strategy facilitated the practical implementation of the FCN role and its associated activities. As one nurse described, *“[Our managers] accepted all our initiatives, all our projects, and allowed us to carry them through to completion. I think that if this has been an oppositional organization, they would not have let us do certain things or given us the opportunity to propose them*.” (FCN_14).

Ministerial Decree 77/2022 also required the introduction of new healthcare services and organizational models, such as Territorial Operational Centers within each region. In Biella LHA, two Territorial Operational Centers were established in 2023 and four FCNs were employed within these centers. These FCNs were carefully selected for their specific education as well as their unique project management skills and commitment to community‐focused initiatives (Table 2_Tailoring strategies). A specific aim of the Territorial Operational Center is to coordinate care across different healthcare settings, playing a pivotal role in formalizing the innovative aspect of the FCN role, such as care coordination, health promotion initiatives, and community education projects (Table 2_Relative priority).

The process of establishing the credibility of the FCN role within the community was multifaceted and required both strategic stakeholder engagement and nursing workflow adaptation. This process began with the active involvement of key community stakeholders, including local authorities, general practitioners, and volunteer organizations (Table 2_Partnership and Connections). Nurse managers organized formal presentations to introduce the FCN role, ensuring that healthcare professionals and community leaders understood its purpose and benefits. FCNs then held individual meetings with stakeholders to tailor community projects to local needs and strengthen collaboration (Table 2_Engaging).

The integration of community interventions into the existing nursing workflow required careful planning. These interventions were meant to be planned by the four FCNs employed within the Territorial Operational Centers and disseminated across the district through the active involvement of other FCNs within the nursing teams (Table 2_Engaging). In the initial phase, it was necessary to optimize resource allocation to ensure nurses’ participation in community education projects. Nurse managers initially attempted to make FCN participation in community events mandatory by incorporating them into nurses’ shifts. However, in implementing this strategy, managers experienced resistance from some FCNs, who perceived that activities required interactions (e.g., with children or in public events) for which they felt unprepared or uncomfortable (Table 2_Tailoring strategies). As one manager explained, *“Initially, we tried to make project participation mandatory. Some people didn’t voice their concerns or outright said, ‘I don’t want to work with children.’ So, we decided to involve everyone, distributing them across different projects”* (M_1).

In response to the resistance from the nurses, the managers developed and advanced a more flexible and inclusive strategy to encourage nurse engagement in the community education projects, this time considering individual nurses’ preferences and skills. Flexible work shifts were also introduced to balance direct care activities (mainly carried out in the morning) with participation in community education projects (primarily held in the afternoon) (Table 2_Tailoring strategies).

Despite the changes adopted to enable nurses to perform their role at the community level, the process remained in a developmental phase. Nurses and managers recognized the need for continuous refinement and piloting of novel strategies to reach a stage where FCNs are seamlessly integrated within these community initiatives. Community interventions were subsequently tested on a small scale and refined based on feedback, reflecting an adaptive approach. As one FCN shared, *“I’ve realized that we’re still at the beginning, so it’s all about trying things out and seeing how they go. This time it worked well here, but we’ll see if it works the same way in another municipality.” (FCN_9)*. This highlights the experimental nature of the process, with a trial‐and‐error approach guiding the refinement of community projects. In addition, at the time of this study, the process still lacked structured evaluation tools, making it challenging to fully assess long‐term impacts and scalability (Table 2_Reflecting and evaluating). As one participant noted, “*We don’t yet know the full impact of our projects. They seem beneficial, but we have no concrete data to support this*” (FCN_16).

### 4.2. Outer Setting Factors Influencing the Adoption of the FCN Role

While legislative frameworks, such as Ministerial Decree 77/2022, have provided structural support for integrating FCNs into community healthcare (Table 2_Policies and Law), their implementation has been hindered by geographical constraints and cultural perceptions of the nursing role. Among the barriers related to the outer setting, both organizational factors linked to territorial morphology and cultural attitudes toward nursing directly influenced the utilization of FCNs by citizens.

One of the main organizational challenges stems from the territorial morphology (Table 2_Local conditions), which includes both large urban centers and rural and mountainous areas. In mountainous areas, population dispersion and the distance between homes and healthcare services make access to care more difficult. Many FCNs operate in small, predominantly elderly communities, where limited public transportation restricts community members’ access to essential services. Working in these contexts requires a significant effort from FCNs, who need to overcome logistical challenges such as traveling long distances and navigating challenging road conditions, particularly during the winter months. Consequently, FCNs adapt their schedules, and care approaches to ensure that even the most isolated individuals receive continuous and equitable assistance. This includes the provision of a wide range of services, from basic nursing care (e.g., wound care, medication administration, and monitoring of vital signs) to health education within community nursing stations, open on specific days and times.

In addition to geographical constraints, cultural attitudes and historical perceptions of nursing roles present another significant outer setting barrier (Table 2_Local attitude). Citizens still associate nurses exclusively with task‐oriented care, rather than viewing them as health educators and community health promoters. This lack of understanding of the FCN role translates into low levels of engagement in community interventions, as citizens struggle to grasp why a nurse would visit them outside of an immediate health concern. As one nurse stated: *“We primarily work with elderly patients, and for them, it doesn’t matter if we’re district nurses or FCNs. They stop at the word ‘nurse’ because what matters to them is their immediate health need, not the type of nurse who provides it. Nurses have always been seen as the ones who give injections or take blood samples*.” (FCN_3). Another participant described how individuals struggle to understand the rationale behind nursing interventions that focus on prevention and health promotion prior to the onset of symptoms of illness, reinforcing the idea that healthcare is only necessary when a problem arises. One nurse summarized this challenge by explaining:

*If it’s a healthcare-related issue, it’s understood and accepted. But if it’s more about assessing social and environmental conditions, there’s no cultural framework to support it. People don’t understand why someone should be doing this kind of work. They don’t know, and even if you explain it to them, it’s difficult for them to grasp. They ask, ‘If I’m fine, why should the nurse come? Why is the nurse here?’ Culturally, it’s a difficult concept for them to understand.* (FCN_2).

### 4.3. Inner Setting

The inner setting encompasses the structural, cultural, and contextual characteristics within the LHA of Biella that shaped the implementation of the FCN role. Several factors—such as a patient‐centered care approach, mentorship and support systems, and established communication channels—were already in place prior to the role’s introduction and influenced the environment into which the FCNs were integrated.

Additionally, throughout the implementation process, new factors emerged within the inner setting, including the predominance of task‐oriented home care demands over community‐level prevention efforts, and the perception among FCNs that engaging in health promotion initiatives involved an additional workload. Together, these preexisting and emergent elements of the inner setting played a critical role in influencing the successful integration and operationalization of the role.

#### 4.3.1. Preexisting Factors in the Inner Setting

For some nurses, who previously worked in hospitals that use primary nursing care models to deliver patient‐centered care, this facilitated the transition into the FCN role. The primary nursing care model, implemented in hospital settings, assigns a single nurse—referred to as the primary nurse or referent nurse—the responsibility for planning and overseeing the patient’s care throughout their entire hospital stay. This approach ensures that, whenever possible, the primary nurse provides direct assistance to the assigned patient, reinforcing continuity of care and fostering a stronger nurse–patient relationship (Table 2_Structural characteristics: work infrastructure). By promoting a patient‐centered approach, FCNs with this background in primary nursing helped these nurses develop a continuity‐of‐care mindset, which closely aligns with Family and Community Nursing principles. As a factor facilitating this transition to the new role, one FCN confirmed that “*What helped me was having done Primary Nursing, where care was more individualized, patient-centered, and slightly extended to the family members—it was already part of my way of working.”* (FCN_10).

Workplace mentoring and peer support also played a crucial role in facilitating the integration of nurses transitioning from hospital settings. The presence of experienced colleagues helped new FCNs orient themselves within the system through structured mentoring processes and knowledge sharing (Table 2_Structural characteristics: work infrastructure and communications). Although structured information‐sharing processes exist within nursing teams, barriers to interdisciplinary collaboration persist, primarily due to rigid professional boundaries and hierarchical organizational structures, which hinder effective information sharing and coordination across teams.

A defining aspect of FCN work is the development of healthcare networks. The purpose of these healthcare networks is to facilitate coordinated, patient‐centered care by fostering collaboration and communication among diverse healthcare professionals and services. A primary and priority focus in the early implementation of the FCN role into the system was to actively engage other professionals within the system—following a horizontal vision of network‐based collaboration—and seek to develop novel strategies to address the traditional fragmentation among healthcare professions. The FCNs’ commitment to interprofessional collaboration was particularly evident in contexts characterized by complex health needs, where the contribution of each professional was essential to ensure complementary and integrated care (Table 2_Relational connections). This perspective is clearly expressed by one nurse:

*Family and Community Nursing is based on healthcare networks, where each professional contributes as if they were a piece of a puzzle. This process takes place collaboratively—through phone consultations and direct discussions. If I encounter a complex issue, I need to work with other professionals: the physiotherapist, the speech therapist, whoever is needed.* (FCN_13).

However, while network‐based collaboration is well‐established among certain professionals, challenges remain in integrating FCNs into hospital‐based services (Table 2_Relational connections). Difficulties in building effective relationships with hospital professionals—such as dietitians—persisted across the different phases of the implementation process. These challenges were rooted in a lack of mutual understanding of professional roles and concerns about role overlap, particularly in health promotion and nutrition education interventions. Although these activities fell within the FCNs’ scope of practice, their contribution was often not fully recognized and was mostly perceived as an intrusion into other professionals’ domains. These tensions were further reinforced by organizational silos, which limited opportunities for cross‐sector collaboration. As a result, FCNs’ ability to establish meaningful partnerships within hospital services remained limited. As one FCN stated:

*In some projects, we have successfully integrated with other professionals, but with some, it is still difficult—even today. These challenges have always existed. Paradoxically, collaborating with municipalities and volunteer organizations seems easier than with some hospital services. We conduct health promotion interventions and discuss healthy eating, but some professionals perceived this activity as an intrusion, as if it were exclusively their domain. We tried to clarify that this topic is part of our nursing competencies. However, the message was not well received, and we continue to face difficulties.* (FCN_14)

#### 4.3.2. Ongoing Challenges Following FCN Role Implementation

Efforts to align with Ministerial Decree 77/2022, combined with the care demands of a population characterized by high levels of aging and chronic conditions, reinforced the perception that task‐oriented activities and family education in home care settings take precedence over community‐level prevention and health promotion initiatives (Table 2_Relative Priority). Although FCNs are trained to implement community‐based projects, these activities have often been given lower priority compared to direct patient care. This tendency has been exacerbated by the challenges of transitioning to a proactive healthcare model. As a result, FCNs’ involvement in health promotion and prevention activities has been systematically limited by the heavy workload associated with home care delivery and competing organizational priorities. Community‐based interventions—such as educational activities in schools or awareness campaigns—often coincide with the planning and management of home care services, making it difficult for FCNs to participate. As one manager explained, *“When projects about promotion are discussed, nurses naturally prioritize organizing home visits, and to avoid generating overtime, sometimes it’s just not possible to give availability for promotion initiatives in the afternoon/evening hours.”* (M_3).

In contexts where the task‐oriented model had historically existed, when the health promotion and prevention activities core to the FCN role were required to be implemented, this latter work was perceived as and experienced as additional workload (Table 2_Adapting). The consequence then was that FCNs found themselves working extra hours to avoid burdening colleagues. As one participant noted: *“I would love to dedicate more time to education and community events, but if I don’t carry out home visits, no one else will do them in my place. So, in order to participate, I have to work outside my regular hours, which means generating overtime. This not only discourages participation but also creates an organizational challenge.”* (FCN_6). As a result, the time allocated to home care was significantly greater than the time and nursing resources dedicated to community‐based interventions. One FCN quantified this imbalance: *“If I had to estimate the time I dedicate to family care versus community activities, I would say that 95% is spent on patients and only 5% on community initiatives.”* (FCN_16).

### 4.4. Individual Factors

The adoption of the FCN role is shaped by an interaction between motivations, opportunities, and capabilities at the level of the individual FCN or manager. Motivation plays a crucial role in the decision to become an FCN and manifests in two main dimensions: the drive to enhance and formalize one’s skills—gained through experience—by acquiring specialized training, and the desire to follow the patient’s care journey (Table 2_Motivation). Hospital‐based nurses seeking to transfer to the district setting emphasized that the transition to community‐based nursing was perceived as an opportunity to ensure continuity of care for patients beyond hospital discharge. For these nurses, the transition represented a professional opportunity to adopt a more holistic approach to patient care and work at their full scope of practice. This perspective underscores the need for a sustained engagement with patients across different care settings. One participant described this necessity as an essential professional requirement: “*What I did in my hospital ward ended there, but it wasn’t complete. I needed to see the patient’s care plan fully realized. I needed to plan and follow through—to understand that, for that person, everything we had planned and done together had led to a tangible benefit. This is what led me to approach Family and Community Nursing*.” (FCN_1).

Within the CFIR framework, capability refers to the degree to which individuals possess the interpersonal competence, knowledge, and skills necessary to fulfill their roles. In the context of implementing the FCN role, both prior supportive leadership and professional experience emerged as critical factors influencing the development of these capabilities.

Managers adopted a supportive leadership style (Table 2_Capability), characterized by active listening and the promotion of continuous professional development, aimed at helping nurses to gain greater confidence and acquire the necessary skills to fully embrace their new role. Despite managerial support, certain FCNs experienced moments of uncertainty, confirming motivation as an essential factor in overcoming difficulties. One nurse emphasized the role of commitment in navigating challenges: “*What kept me going (during difficult times) was my strong belief in this role […] I am glad I persevered because I know I can make a difference for both the patient and their family.”* (FCN_7).

Beyond motivation, prior work experience significantly contributed to individual capabilities, facilitating the adoption of the FCN role (Table 2_Capability). Nurses transitioning from hospital settings brought with them robust clinical expertise and well‐developed assessment skills, enhancing their ability to manage complex situations and evaluate the health and social circumstances of patients and their families. This experiential background strengthened their capacity to navigate interdisciplinary care pathways. Conversely, participants with less work experience faced challenges in fully embracing the preventive and community‐oriented aspects of the role. As one FCN stated: “*I still feel a little unprepared*.” (FCN_10).

## 5. Discussion

This qualitative case study described the implementation of the FCN role within the LHA of Biella, Northern Italy. Drawing on the CFIR framework [24], the analysis identified contextual, organizational, and individual factors that influenced this process. Key findings highlighted how legislative support, geographical and cultural barriers, preexisting organizational structures, and individual motivations and capabilities shaped the success of FCN role adoption.

The implementation of a new professional role within an existing healthcare system represents a complex intervention, characterized by multiple interacting components and the need for adaptation across diverse settings and stakeholders. The CFIR’s five domains [24]—intervention characteristics, outer setting, inner setting, characteristics of individuals, and the implementation process—enabled a theory‐informed and nuanced examination of the dynamic evolution of the FCN role. This framework was particularly valuable in capturing the iterative nature of implementation, highlighting how barriers and facilitators shifted over time in response to contextual changes, professional engagement, and organizational learning.

The implementation process described in this case study reflects an ongoing expansion of the FCN role along three key dimensions: (1) a shift from a task‐based model to one centered on care coordination, education, and prevention; (2) a reorientation of delivering care from the individual and family to the broader community; (3) and a movement from a disease‐focused to a person‐centered, holistic approach.

### 5.1. Shifting From Task‐Based Care to Coordination, Prevention, and Education

The transition from hospital‐based, task‐oriented nursing to community‐based roles centered on health promotion, care coordination, and health education represents a significant paradigm shift in nursing practice in Italy. Existing literature highlights that this shift requires nurses to adopt broader, preventive, and population‐focused approaches, emphasizing relationship‐building, interprofessional collaboration, and responsiveness to social determinants of health. Karam et al. [28] found that care coordination by nurses in primary healthcare settings, particularly for patients with complex needs, is most effective when nurses are given clear role definitions, supportive interprofessional relationships, and sufficient organizational infrastructure. However, role ambiguity and limited system‐level understanding can impede full integration, particularly in settings still dominated by task‐based models of care. These findings reinforce the importance of organizational support, leadership, and capacity‐building to enable nurses to operationalize expanded roles and improve access, continuity, and quality of care.

In parallel with the professional challenge faced by nurses in transitioning toward a less task‐oriented role, this case study also revealed the difficulty of reshaping how nursing roles are perceived by others. Socially, within this Italian context, there is a persistent perception of nurses as task‐oriented professionals, primarily responsible for providing technical interventions such as medication administration or wound care. This limited view hinders the recognition of this new role of FCNs as autonomous professionals with a broader mandate that includes care coordination, prevention, and community‐based health initiatives. A significant and consistent barrier to the development of advanced nursing roles in primary care lies in the lack of clarity and recognition regarding their scope of practice and competencies [20, 29]. This case study reinforces that observation, revealing how both citizens and other health professionals continue to struggle with understanding the added value of FCNs in care continuity and public health initiatives. To address this cultural and professional ambiguity, it becomes essential to actively promote the recognition of FCNs as pivotal actors in the primary care system. Changing public and professional perceptions is not merely a matter of raising awareness; it also requires long‐term institutional strategies. These should include targeted communication efforts, interprofessional education, and structural interventions aimed at consolidating the FCN role within a more integrated and proactive model of care, as well as fostering citizen engagement by addressing the cultural and practical barriers that often hinder participation in community‐based health initiatives.

Promoting the social and professional recognition of FCNs calls for institutional campaigns and the development of interprofessional training pathways aimed at fostering a shared language and common operational practices among primary care professionals. In line with a Population Health Management approach [30], such training should support proactive, data‐driven, and person‐centered models of care, helping to overcome fragmentation among care providers and reinforcing the FCN’s visibility and legitimacy to both colleagues and citizens. Recent experiences have shown how a Population Health Management approach can enhance interprofessional collaboration, support shared decision‐making, and foster strategic alignment, key conditions for the effective integration and recognition of advanced roles like the FCN [31, 32].

### 5.2. Shifting Care Delivery From Individuals to Communities

The second key shift in nursing practice involves a reorientation from an individual‐ and family‐centered model of care to one that embraces the broader community as its primary focus. This transition reflects a growing recognition that addressing social determinants of health, promoting prevention, and supporting population‐level health outcomes require nurses to act beyond the boundaries of individual clinical encounters [33]. The FCN role is specifically designed to support this broader mission, yet its translation into practice remains uneven, especially in contexts where the role has been recently introduced. In an Austrian study of 130 community health nurses involved in national pilot projects, nurses mostly operated at the individual level despite their broader community‐oriented mandate [34]. This is paralleled in our study, where community‐based interventions often remain marginal in day‐to‐day nursing practice, as task‐oriented demands in home care continue to dominate work schedules. As shown in these findings, nurses themselves recognize this imbalance and express a desire to fully enact the FCN scope of practice but face structural and cultural constraints that inhibit immediate change. Although not raised directly by participants, the nationally reported FCN‐to‐inhabitant ratio [14] further illustrates the gap between the broader community‐oriented expectations of the FCN role and the reality of everyday practice. Such systemic conditions make it difficult for FCNs to dedicate time to prevention, health promotion, and community engagement, thereby reinforcing the predominance of task‐oriented work observed in this study.

### 5.3. Shifting From Disease‐Focused to Person‐Centered Care

The third shift in nursing practice reflects a gradual transition from a disease‐focused model of care to one that is person‐centered and holistic. Rather than focusing on discrete clinical problems, FCNs increasingly aim to engage with the full spectrum of individuals’ and families’ life situations, values, and evolving care needs. This approach reflects a core tenet of advanced nursing practice [35]: integrating clinical expertise with relational, observational, and reflective competencies to support health and well‐being in a more comprehensive way. However, the findings reveal a persistent tension between this holistic intent and the structural constraints of daily practice. Workload pressures, performance metrics tied to technical tasks, and a prevailing biomedical culture can limit the time and space available for meaningful, person‐centered encounters. Despite these challenges, FCNs in this study demonstrated a strong commitment to building trusting relationships, conducting in‐depth assessments, and responding to the unique circumstances of the people they serve—often going beyond formal protocols to address unspoken needs.

The expansion of the FCN role along these three key dimensions, as depicted in this case study, is aligned with global policy directions that advocate for strengthening primary healthcare through advanced nursing roles [36]. Evidence from OECD countries with longer standing experience in implementing advanced practice nursing roles in primary care—such as the United States, Canada, and Australia—demonstrates consistently positive outcomes associated with their integration [37]. These include improved access to care, smoother transitions across care settings, reduced hospital (re)admissions, and higher patient satisfaction, particularly when nurses are adequately trained and supported to work to their full scope of practice. In Italy, the National Nurses’ Regulatory Body has promoted the development of the FCN role through advanced training pathways at master’s level aligned with international standards [35], with the aim of enabling role expansion and formal integration into primary care. However, as of 2025, this role has not yet been formally recognized in the national regulatory framework [37].

Within this evolving context, the FCN role can be compared with advanced practice nursing roles such as Clinical Nurse Specialists in terms of future directions rather than the current scope of practice. Unlike Clinical Nurse Specialists, FCNs do not perform medical diagnoses, prescribe treatments, or provide advanced disease‐specific clinical management. Instead, the FCN role, as currently implemented, enacts advanced nursing practice through care coordination, health education, community engagement, and the integration of services, with a strong orientation toward public health and population‐level needs. The FCN role is however particularly relevant to addressing key health system challenges in Italy, including population aging, multimorbidity, frailty, and the need for improved continuity across primary, community, and hospital care.

This case study also highlights how managerial leadership styles grounded in collaboration and shared governance enabled the successful implementation of the FCN role [38]. Although nurse managers did not formally adopt a shared decision‐making framework, they consistently engaged nurses in shaping the role’s development—soliciting input on community projects, valuing their specialized training, and adapting work schedules to support new activities. These practical, inclusive approaches reflect relational and transformational leadership styles, which emphasize trust, empowerment, and responsiveness to team needs. Such leadership practices have been identified as essential in supporting role clarity, professional autonomy, and innovation in nursing [39]. In this context, investments in open communication, mentorship, and ongoing professional development were not ancillary supports but core conditions that enabled the FCN role to take root and evolve within the local health system.

While managerial leadership played an important role in supporting the implementation process, these findings also call attention to another critical dimension: clinical leadership exercised by FCNs themselves. Another important implication of this case study is the leadership potential of FCNs, particularly in the realm of public and population health. Participants described some examples of FCNs initiating and leading community‐based projects, despite limited resources or formal mandates. These efforts demonstrate that FCNs can act as leaders in developing and delivering health promotion strategies tailored to local needs. However, especially in the Italian context, nursing leadership has often been narrowly interpreted as relevant only to managerial roles, overlooking its expression in direct care settings [40]. Supporting clinical leadership among FCNs requires not only integrating leadership development into professional education but also fostering organizational cultures that empower nurses to lead change [41]. International literature highlights the importance of clinical leadership in achieving better health outcomes, strengthening interdisciplinary collaboration, and fostering innovation in care delivery [42].

This is the first Italian case study aiming to describe the implementation of the FCN role using the CFIR. However, there are a small number of additional studies that have studied various aspects of the processes to adopt, implement, and integrate the FCN role across different geographic regions in Italy. Taddeucci et al. [43] showed how the same regional policy was variably interpreted across three Tuscan LHAs, influenced by local organizational histories. Scrimaglia et al. [44] highlighted unmet needs and the potential of FCNs in rural Emilia‐Romagna, emphasizing interprofessional collaboration. Clodig et al. [45] reported a participatory codesign process in Friuli Venezia Giulia to define FCN responsibilities. Our findings complement these contributions by detailing how local leadership, contextual tailoring, and adaptive strategies shaped implementation, and by identifying key organizational and cultural enablers and barriers. Applying CFIR allowed for a structured understanding of the implementation process, which may inform future scaling efforts across diverse Italian contexts.

## 6. Limitations and Strengths

This study explores the implementation of the FCN role in a single LHA, employing the CFIR framework. Triangulation of two unique data sources, nursing managers and FCNs, enabled an in‐depth and multiperspective understanding of organizational challenges, generating practical insights directly applicable to daily clinical practice. Coding, categorizing, and synthesis of all data were completed by two nurse researchers, which promoted the overall dependability of the findings. Additionally, the use of a robust conceptual framework to structure the analysis process, novel within the Italian context, facilitates international comparisons and provides useful insights for external readers. A further strength is that all interviewed FCNs were observed during the immersion period prior to their interviews, which enhanced the credibility of their accounts by anchoring them to directly observed practice. However, the study has several limitations. It predominantly focuses on the initial and intermediate phases of FCN implementation, thus lacking an assessment of the long‐term sustainability of the role. The absence of nurses with complementary roles (e.g., territorial wound‐care specialists) and other relevant professional stakeholders (such as GPs and social workers) restricts a comprehensive understanding of interprofessional collaboration and the scope of the FCN role compared to established primary care nursing models. Finally, the retrospective nature of data collection may have introduced potential inaccuracies in participants’ recall of past experiences and perceptions. Although FCNs entered the role in different years, the use of timelines during the interviews helped anchor their accounts to specific implementation events, thereby mitigating potential inaccuracies in temporal reconstruction.

## 7. Conclusions

Implementing the FCN role is a dynamic and context‐sensitive process, shaped by system structures, professional boundaries, and stakeholder engagement. This study showed how organizational flexibility and supportive leadership enabled local adaptation, while structural constraints and limited awareness of the role hindered full integration. Ensuring sustainability requires a shared understanding of the FCN’s scope, investment in interprofessional education, and supportive organizational environments. For clinical practice, these findings underscore the need to foster role clarity, encourage collaboration across care settings, and support nurses in exercising clinical leadership—particularly in the areas of prevention, health promotion, and care coordination for aging populations. In addition, further research is needed to evaluate the extent to which the FCN role is able to address the evolving complexity of population health needs.

## Author Contributions

All authors have made substantial contributions to the conception and design, acquisition of data, analysis and interpretation of data, drafting of the article, and revising it critically for important intellectual content.

Susan M. Jack and Alberto Dal Molin are co‐last authors and contributed equally to this work.

## Funding

This study is part of the project Age‐It, which has received funding from the MUR—M4C2 1.3 of PNRR funded by the European Union—NextGenerationEU (Grant agreement no. PE_00000015). Open access publishing facilitated by Universita degli Studi del Piemonte Orientale Amedeo Avogadro, as part of the Wiley ‐ CRUI‐CARE agreement.

## Disclosure

All authors have agreed on the final version.

## Ethics Statement

Ethics approval was obtained from the Local Ethics Committee (Approval No. CE 104/2024).

## Consent

All participants gave written informed consent before taking part in the interviews, in accordance with the Declaration of Helsinki and institutional ethical guidelines.

## Conflicts of Interest

The authors declare no conflicts of interest.

## Data Availability

The data that support the findings of this study are available from the corresponding author upon reasonable request.
